# Clinical variations of polypoidal choroidal vasculopathy: A cohort study from Japan and the USA

**DOI:** 10.1038/s41598-023-31649-x

**Published:** 2023-03-23

**Authors:** Hisashi Fukuyama, Ghazi BouGhanem, John Moir, Dimitra Skondra, Fumi Gomi, Amani A. Fawzi

**Affiliations:** 1 Department of Ophthalmology, Feinberg School of Medicine, Northwestern University, Chicago, IL USA; 2 Department of Ophthalmology, Hyogo Medical University, 1-1 Mukogawa-cho, Nishinomiya, 663-8501 Japan; 3 Department of Ophthalmology and Visual Science, The University of Chicago, Chicago, IL USA; 4 Department of Ophthalmology, Feinberg School of Medicine, Northwestern University, 645 N. Michigan Ave., Suite 440, Chicago, IL 60611 USA

**Keywords:** Medical research, Health care

## Abstract

We describe the clinical characteristics of treatment-naïve polypoidal choroidal vasculopathy (PCV) in three tertiary clinic settings in 2 cities (Chicago in the USA and Nishinomiya in Japan). This cohort study was a retrospective, multicenter, consecutive case series. A total of 126 patients with treatment-naïve PCV—46 in Chicago and 80 in Nishinomiya—were identified. The proportion of PCV in patients with neovascular age-related macular degeneration was lower in Chicago (10.8% vs. 36.9%). Patients in Chicago had a significantly higher prevalence of soft drusen (50.0% vs 25.0%, p = 0.006) and intra-retinal cyst (37.0% vs 15.0%, p = 0.008), and a significantly lower prevalence of pachyvessels (41.3% vs 62.5%, p = 0.03). At baseline, presenting vision for patients in Chicago was worse than in Nishinomiya (mean log MAR: 0.609 vs. 0.312, p < 0.001). Ninety-five eyes were followed for more than one year. The Nishinomiya group received a higher rate of combination therapy (61.0%) compared to the Chicago group (5.3%). Vision and central foveal thickness at month 12 were significantly improved from baseline in both Chicago (p = 0.009 and p = 0.01) and Nishinomiya groups (both p < 0.001). Our study highlights interesting differences in the proportion of PCV, clinical findings and treatment responses of PCV, that need to be further evaluated in larger, epidemiologic cohorts.

## Introduction

Age-related macular degeneration (AMD) is a progressive chronic disease and a leading cause of blindness in elderly people. The prevalence of AMD increases with age^1^. AMD accounts for 8.7% of all blindness worldwide and is the most common cause of blindness in developed countries. The estimated population with AMD worldwide is projected to reach 288 million by 2040^2^. Polypoidal choroidal vasculopathy (PCV) is considered a subtype of AMD, associated with recurrent serous subretinal fluid and subretinal hemorrhage, vitreous hemorrhage, and relatively minor fibrotic scarring compared with typical AMD^3^. PCV has also been considered to be one of the pachychoroid spectrum diseases, characterized by similar choroidal findings, such as diffuse or focal areas of increased choroidal thickness and dilated choroidal vessels^4^. According to recent interpretation, PCV is likely multifactorial, with features that overlap with AMD as well as pachychoroid diseases^5^.

The etiology of PCV remains largely unknown. PCV reports in Black and Asian patients are more frequently seen in the literature, and both these racial and ethnic groups have a higher risk of developing PCV compared to White patients^6,7^. Although previous data vary widely in different countries, and most large PCV studies arise from Asian countries^8–10^. However, recent research suggests that PCV is not rare in White patients, who present with occult choroidal neovascularization^11,12^, and the presentation of White patients is more closely related to typical neovascular AMD^13,14^. The current gold standard for diagnosing PCV requires indocyanine green angiography (ICGA), but this is a relatively invasive procedure that is not routinely performed in eyes with exudative AMD, which could account for a relative underdiagnosis of PCV in specific environments where ICGA is unavailable or unpopular.

Clinical characteristics and treatment responses of PCV are highly variable due to differences in etiology, genetics, and morphology^15–17^. A variety of different subtypes of PCV have been suggested^18^, prompting novel classification systems^19^. Whereas intravitreal injections of anti-vascular endothelial growth factor (anti-VEGF) agents represent the standard of care for neovascular AMD, some PCV lesions respond less favorably to anti-VEGF monotherapy, and this could be caused by the substantial variation in the population^20^. In fact, substantial variation in PCV has been reported in terms of demographic characteristics, risk factors, genetic association, clinical presentation, and natural history in different populations^21–23^.

In this study, we proposed to compare the clinical presentation of PCV in three tertiary referral institutions in two different cities in different countries (Chicago in the USA and Nishinomiya in Japan). In addition, we also proposed to evaluate the difference in clinical presentation among patients in these different settings. Therefore, we discuss the difference in clinical characteristics, treatment algorithms and treatment responses of PCV.

## Results

### Demographic and clinical characteristics difference between Chicago and Nishinomiya

**Figure 1:**
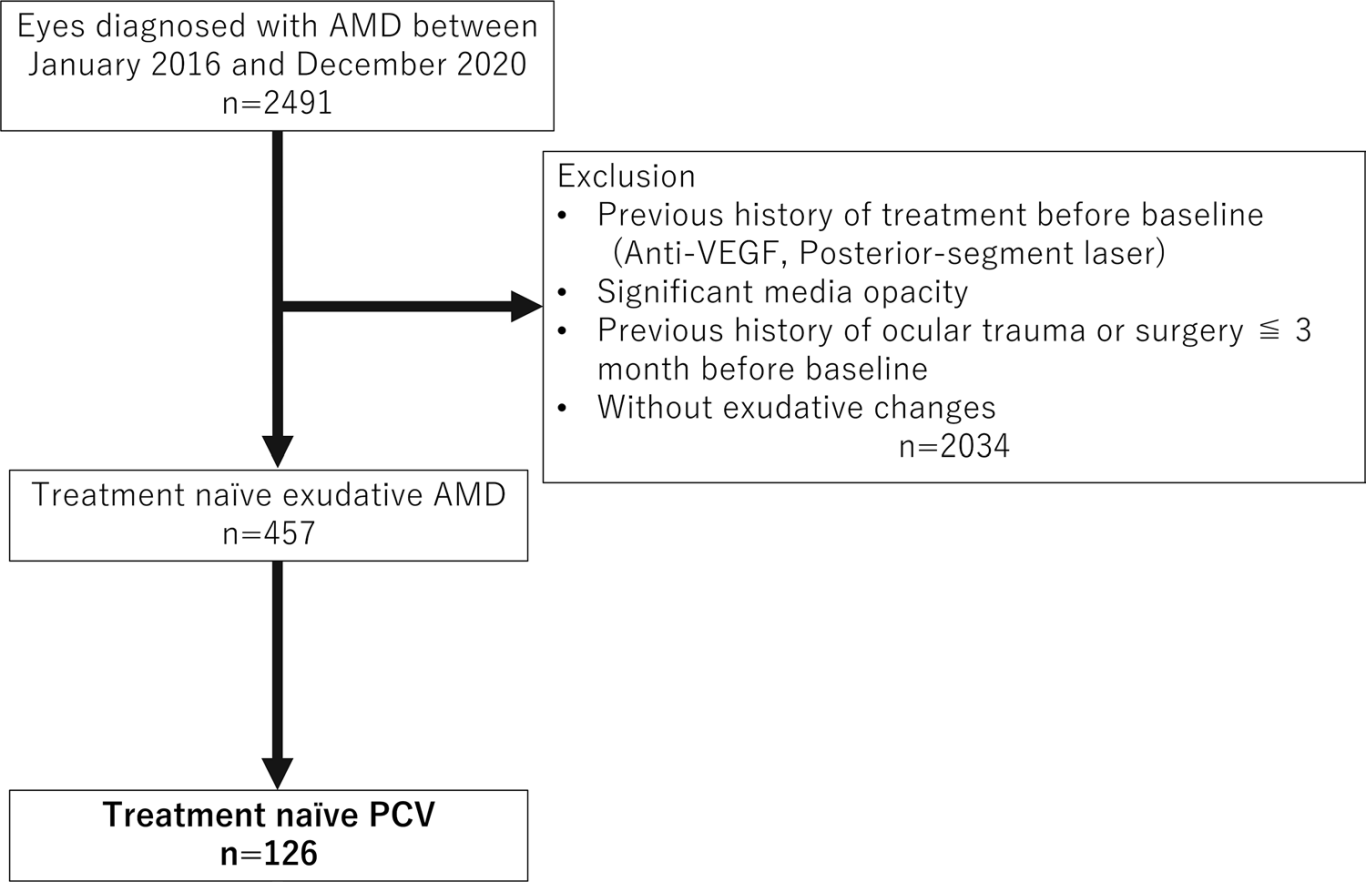
Flowchart of our retrospective study design. *AMD* age-related macular degeneration, *PCV* polypoidal choroidal vasculopathy.

A flow chart of the patient selection process is shown in Fig. 1. We reviewed 2491 individuals with AMD, and 457 individuals were diagnosed with treatment-naïve exudative AMD. Of these individuals, 126 were confirmed as having PCV based on the optical coherence tomography (OCT) criteria. The proportion of PCV in patients with treatment-naïve exudative AMD was 19.7% (10.8% in Chicago, 36.9% in Nishinomiya). Table 1 shows the demographic characteristics of the entire study population. Seventy-five patients were men, 51 patients were women, and the mean age was 73.8. The Chicago group had a higher percentage of females (69.6%), and the Nishinomiya group had a lower body mass index (BMI) (mean: 23.1 kg/m^2^).

**Table 1:** Baseline demographic characteristics for Chicago and Nishinomiya patients.

	Total (n = 126)	Chicago (n = 46)	Nishinomiya (n = 80)	p
Age (years)	73.8 ± 9.5	73.3 ± 11.3	74.2 ± 8.3	0.92^a^
Gender (male) (%)	75 (60.0%)	14 (30.4%)	61 (76.3%)	< 0.001^b^
BMI	24.7 ± 4.4	27.4 ± 5.2	23.1 ± 3.0	< 0.001^a^
Smoker (current or former) (%)	62 (49.2%)	25 (54.4%)	37 (46.3%)	0.46^b^
DM (%)	17 (13.5%)	8 (17.4%)	9 (11.3%)	0.41^b^
HTN (%)	75 (59.5%)	30 (65.2%)	45 (56.3%)	0.35^b^
Ischemic cardiac disease (%)	12 (9.5%)	3 (6.5%)	9 (11.3%)	0.53^b^
Renal disease (%)	3 (2.4%)	2 (4.4%)	1 (1.3%)	0.55^b^

^a^Mann–Whitney *U* test and ^b^Fisher’s exact test were used to calculate p values.

*Significant at p < 0.05.

*BMI* body mass index, *DM* diabetes mellitus, *HTN* hypertension.

Table 2 shows the clinical characteristics of the study population. One hundred twenty-six eyes in 126 patients were included. The prevalence of soft drusen and intra-retinal cysts was significantly higher in the Chicago group (50.0% and 37.0%, respectively) compared to the Nishinomiya group (25.0% and 15.0%, respectively; p = 0.006 and p = 0.008). The Chicago group had a lower percentage of pachyvessels (41.3%) compared to the Nishinomiya group (62.5%). Mean baseline visual acuity (VA) was 0.609 in Chicago and 0.312 in the Nishinomiya groups, significantly worse in Chicago (p < 0.001). The prevalence of subretinal hemorrhage was 41.3% (52/126 eyes). Among these, the prevalence of subretinal hemorrhage involving the fovea was 88.5% (46/52 eyes). The subfoveal choroidal thickness (SFCT) in the Nishinomiya group was significantly larger than the Chicago group (237 vs. 195 um, p = 0.011). SFCT in fellow eyes (mean, 221 um) was significantly lower than SFCT in the affected eye (p = 0.002).

**Table 2:** The baseline clinical characteristics for Chicago and Nishinomiya patients.

	Total (n = 126)	Chicago (n = 46)	Nishinomiya (n = 80)	p
Eyes (right) (%)	51 (40.5%)	23 (50.0%)	28 (35.0%)	0.13^a^
Location of polypoidal lesion				0.19^b^
Macular (%)	91 (72.2%)	33 (71.7%)	58 (72.5%)	
Peripapillary (%)	23 (18.3%)	11 (23.9%)	12 (15.0%)	
Other (%)	12 (9.5%)	2 (4.4%)	10 (12.5%)	
VA	0.421 ± 0.457	0.609 ± 0.562	0.312 ± 0.340	< 0.001^c^
Hard exudates (%)	40 (31.8%)	13 (28.3%)	27 (33.8%)	0.56^a^
Soft drusen (%)	43 (34.1%)	23 (50.0%)	20 (25.0%)	0.006^a^
Pachydrusen (%)	44 (34.9%)	13 (28.3%)	31 (38.8%)	0.25
Subretinal hemorrhage (%)	52 (41.3%)	21 (45.7%)	31 (41.3%)	0.50^a^
Intra retinal fluid (%)	29 (23.0%)	17 (37.0%)	12 (15.0%)	0.008^a^
Pachyvessels (%)	69 (54.8%)	19 (41.3%)	50 (62.5%)	0.03^a^
Double-layer sign	67 (53.2%)	20 (43.5%)	47 (58.8%)	0.14
CFT (um)	333 ± 146	327 ± 177	336 ± 126	0.22^c^
SFCT (um)	242 ± 101	233 ± 73	247 ± 115	0.89^c^
Fellow eye				
Pachychoroid disease (%)	28 (21.8%)	10 (21.7%)	18 (22.5%)	1.0
AMD (%)	15 (12.6%)	6 (28.6%)	8 (10.0%)	
CFT (fellow eye) (um)	198 ± 53	194 ± 45	200 ± 57	0.91^c^
SFCT (fellow eye) (um)	221 ± 85	195 ± 67	237 ± 91	0.01^c^

^a^Fisher’s exact test, ^b^Pearson’s chi-square test and ^c^Mann–Whitney *U* test were used to calculate p values.

*Significant at p < 0.05.

*VA* visual acuity, *CFT* central foveal thickness, *SFCT* subfoveal choroidal thickness, *AMD* age-related macular degeneration. *A* Asian, *B* Black, *W* White.

Fifty-two eyes (41.3%) had subretinal hemorrhage at presentation, with subretinal hemorrhage involving the fovea in 46 of 52 eyes (88.5%). Table 3 shows the odds ratio (OR) of subretinal hemorrhage according to demographic characteristics. In the univariate analysis, smoking status (OR 3.10, p = 0.003), Diabetes Mellitus (OR 3.04, p = 0.04) were associated with higher odds of subretinal hemorrhage. In multivariate analysis, only smoking status remained significant. (OR 2.82, p = 0.01).

**Table 3:** Univariate and multivariable analysis of demographic risk factors for subretinal hemorrhage.

Risk factor	OR	95% CI	p (univariate)	OR	95% CI	p (multivariate)
Age	1.02	0.98–1.06	0.30			
Gender (male)	1.32	0.64–2.74	0.45			
Smoker (current or former) (yes)	3.10	1.48–6.50	0.003*	2.82	1.23–6.45	0.01*
BMI	0.92	0.84–1.01	0.07	0.95	0.86–1.05	0.30
DM (yes)	3.04	1.04–8.84	0.04*	2.55	0.74–8.85	0.14
HTN (yes)	1.32	0.64–2.74	0.45			
Ischemic cardiac disease (yes)	1.02	0.30–3.40	0.97			
Renal disease (yes)	0.70	0.06–7.99	0.71			

*Significant at p < 0.05.

*CI* Confidence interval, *OR* odds ratio, *BMI* body mass index, *DM* diabetes mellitus, *HTN* hypertension.

### Time course and clinical outcomes

**Figure 2:**
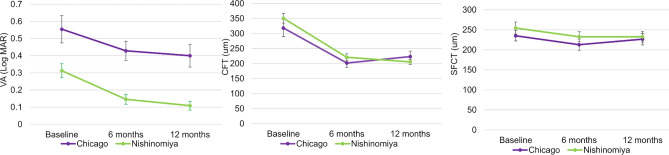
Time course of visual acuity (VA), central foveal thickness (CFT), and subfoveal choroidal thickness (SFCT) between Chicago and Nishinomiya. Vertical bars represent mean ± standard error.

Ninety-seven eyes from 97 patients were followed for more than one year. Thirty-eight of 46 (82.6%) patients in Chicago group and 59 of 80 (73.8%) patients in Nishinomiya group were included in the analysis. Fifty-nine eyes were treated by anti-vascular endothelial growth factor (VEGF) monotherapy, and 38 eyes were treated by combination therapy using photodynamic therapy (PDT) and anti-VEGF. Most eyes (90.4%) were treated with aflibercept (Table 4). In this study, the Nishinomiya group received a higher rate of combination therapy (61.0%) compared to the Chicago group (5.3%). Moreover, the Nishinomiya group received a lower number of anti-VEGF injections (mean: 3.5/year) compared to the Chicago group (mean: 7.6/year). Nishinomiya group had significantly better baseline VA (P = 0.005) (Supplementary table 1). VA and CFT at month 12 were significantly improved from baseline in both Chicago (p = 0.009 and p = 0.01) and Nishinomiya groups (both p < 0.001) (Fig. 2).

**Table 4:** Treatment approach and number of anti-vascular endothelial growth factor (anti-VEGF) injections visits 1 year in patients with Chicago and Nishinomiya.

	Total (n = 97)	Chicago (n = 38)	Nishinomiya (n = 59)	p
Treatment				< 0.001^b^
Anti-VEGF monotherapy	59 (60.8%)	36 (94.7%)	23 (39.0%)	
Combination therapy	38 (39.2%)	2 (5.3%)	36 (61.0%)	
Number of Anti-VEGF (1 year)		7.6 ± 3.4	3.5 ± 2.1	< 0.001^a^

^a^Kruskal–Wallis test and ^b^Fisher’s exact test were used to calculate p values.

*Significant at p < 0.05.

### Racial and ethnic differences

Of the 46 patients with PCV in Chicago, 18 were Black, 21 were White, 4 were Asian, and 3 were Hispanic. All 80 patients with PCV in Nishinomiya were Japanese. The proportion of PCV in Whites was 6.9%, 33.3% in Blacks, and 36.9% in Japanese. Compared to the Nishinomiya group (Japanese group), the prevalence of subretinal hemorrhage was considerably higher in the Black group (72.0%) and lower in the White group (33.3%). VA was worse in the Black group at baseline (mean Log MAR: 0.793). (Supplementary Tables 2 and 3).

For 12 months follow-up (Black: 11 eyes, White: 20 eyes, Japanese: 59 eyes), VA at month 12 was significantly improved from baseline in the Black (p = 0.012) and Japanese groups (p < 0.001). Though the White group received a higher number of anti-VEGF injections, VA at 12 months was not improved from baseline (p = 0.86) (Supplementary Table 4 and Supplementary Fig. 1).

## Discussion

In the present study, we observed several important differences in clinical characteristics and treatment outcomes of PCV between patients who presented to tertiary referral retina clinics in Chicago (USA) and Nishinomiya (Japan). The proportion of PCV was lower in the Chicago group. Clinical characteristics of PCV were also different between the two groups. The presenting vision in the Chicago group was worse than in the Nishinomiya group. On image analysis, compared to the Nishinomiya groups, the Chicago group had less pachyessels, thinner choroid, and more soft drusen and intra-retinal cyst.

The gold standard approach to diagnosing PCV requires the use of ICGA. However, it is not routinely performed in eyes with exudative AMD in many clinical environments, including the USA. This could account for a relative underdiagnosis of PCV in certain populations where ICGA is unavailable or unpopular. The Asia–Pacific Ocular Imaging Society PCV Workgroup recently proposed using non-ICGA diagnostic imaging to manage PCV worldwide^24,25^. Therefore, in this study, we used non-ICGA diagnosis criteria. Using this approach, we found that the proportion of PCV in the overall clinic-based treatment-naïve exudative AMD population was lower in Chicago than in Nishinomiya (10.8% vs. 36.9%). In addition, our study shows that Blacks in Chicago (33.3%) seem to have a higher risk of developing PCV compared to Whites in Chicago (6.9%). Previous studies suggested that the incidence of PCV in Asian patients with neovascular AMD was between 22.3–61.6%^26^ and 4–9.8% in White patients with neovascular AMD^27^. Our data show similar proportion of PCV to these previous studies and adds that similar to Japanese, Blacks in Chicago also have a relatively higher proportion of PCV.

In our cohort, patients in Chicago had significantly higher prevalence of soft drusen (50.0% vs 25.0%) and intra-retinal cyst (37.0% vs 15.0%), lower percentage of pachyvessels (41.3% vs. 62.5%) and SFCT (mean:195 vs. 237 mm) compared to the Nishinomiya population. Recent studies suggested that PCV can be further sub-classified according to the presence of either pachychoroid or AMD features^15–17^. These two subtypes of PCV present differently in terms of their angiographic and tomographic manifestations. Pachychoroid PCV is associated with choroidal vascular hyperpermeability, presence of branching vascular networks, and greater subfoveal choroidal thickness, whereas AMD-feature PCV is associated with soft drusen, intraretinal fluid, and fibrosis^15,28^. The underlying hypothesis is that there are differences in etiology, genetics, and morphology between the two subtypes^13,22^. In this study, the presentation of PCV in the Chicago group is more associated with the characteristics of AMD-features.

In our study, the presenting vision and treatment outcomes were also different between the Chicago and Nishinomiya groups. Presenting vision was worse in the Chicago group at baseline (mean Log MAR: 0.609) than in the Nishinomiya group (mean Log MAR: 0.312). Besides differences in the feature of PCV, potentially these patients had delayed presentation, which could explain the worse presenting vision. In the USA healthcare service, various factors, such as lower incomes^29^, racial and ethnic disparities^30,31^, and geographic access^32^, affect the patient access to ophthalmology, while in Japan health insurance system provides universal coverage and visual impairment is not associated with income level^33^. The difference in health care services and social background could affect the timing of the presentation and presenting vision. Though anti-VEGF is the first-line therapy for PCV in the USA and Japan, in this study, more than 60% of patients in Nishinomiya were treated with a combination of anti-VEGF and PDT therapy, while the Chicago patients were almost exclusively treated with anti-VEGF monotherapy (94.7%). Nevertheless, VA and CFT at month 12 significantly improved from baseline in both Chicago and Nishinomiya groups. On the other hand, when evaluating our data based on racial and ethnic subgroups, VA and CFT did not significantly improve in White individuals at 12 months, despite frequent dosing of anti-VEGF (mean: 8.2). We suspect that the different baseline phenotypes of PCV might be associated with the treatment outcomes. The differences in treatment approaches in the different health systems could also potentially account for our results. Clinical trials using aflibercept have shown that anti-VEGF monotherapy was non-inferior to combination therapy^34,35^. Recent studies have shown that the response to PDT and anti-VEGF drugs might differ according to the choroidal phenotype^16,17^. In a small retrospective study, PDT was more effective in maintaining VA in pachychoroid-feature PCV compared with non-pachychoroid-feature PCV^16^. Along the same lines, another small retrospective study showed that treatment response to anti-VEGF monotherapy was different between pachychoroid-feature PCV and non-pachychoroid-feature PCV. This study showed that pachychoroid-feature PCV had a relatively worse response to anti-VEGF monotherapy compared to AMD-feature PCV^17^. However, these studies included only Asian subjects in the same countries. Therefore, further studies are needed to assess anti-VEGF and/or PDT treatment responses according to choroidal phenotype in different populations. In addition, baseline clinical characteristics, including baseline VA, were different among the racial and ethnic groups. These baseline differences could have influenced the differences in treatment outcomes.

Hemorrhagic complications occur more frequently in PCV than in AMD^3^, and often result in chorioretinal atrophy and poor visual prognosis^18,36,37^. Interestingly, in our cohort, the prevalence of subretinal hemorrhage was not different between Chicago and Nishinomiya groups overall. However, when we evaluated the local prevalence in Chicago, we found that subretinal hemorrhage was more prevalent in the Black group (72.0%) and considerably less prevalent in the White in Chicago (33.3%). The presence of subretinal hemorrhage might be influenced by additional features related to the subject’s environmental and medical background. Therefore, we also evaluated the demographic risk factors that were associated with subretinal hemorrhage and found that smoking was a significant risk factor. Cigarette smoking is a significant risk factor for AMD and PCV^38,39^, and increases the risk of vascular diseases and major bleeding^40,41^. Smoking may increase the risk of polyp rupture and subsequent bleeding in PCV.

There are several limitations to this study. First, we reported a relatively small number of patients in Chicago compared to Nishinomiya, and our study patients were recruited only from three, tertiary referral clinical sites. Second, our study is susceptible to ascertainment bias because of its retrospective design and images obtaining in different OCT machines and protocols. Many aspects of social background, cultural background, and health care services differ across countries, cities, and regions. These differences may influence the timing of the first visit and demographic characteristics. Furthermore, these differences in baseline patient characteristics could have ultimately influenced the treatment outcome. In addition, because of its retrospective study, different treatment regimens in the different hospitals could have affected the treatment outcomes. Moreover, we confirmed the diagnosis of PCV using OCT criteria, while the current gold standard for diagnosing PCV requires ICGA.

In conclusion, the proportion of PCV and clinical findings were different, suggesting pathophysiological differences in PCV among the different populations. We also found that presenting vision and treatment response was different among different populations, which may reflect underlying differences in pathophysiology, treatment algorithms, and/or access to healthcare and timing of presentation. Further prospective studies using the multimodal devices, including ICGA and OCTA, are needed to assess these differences and long-term outcomes in different populations. These studies are needed to help elucidate the utility and relative benefits of anti-VEGF and/or PDT in the management of PCV in different populations.

## Methods

### Participants and methods

This is a multicenter, retrospective cohort study of patients with treatment naïve PCV at the following three institutions: Northwestern University, University of Chicago, and Hyogo Medical University, between January 2016 and December 2020, inclusive. This study was approved by the Institutional Review Board of Northwestern University (approval number STU00214158) and the local institutional review boards of all other participating centers (Hyogo Medical University and University of Chicago), which included a waiver for the requirement of informed consent due to the retrospective nature of the study. In accordance with the IRB approvals, all the patient data in this study were anonymized before analysis. All methods were conducted in accordance with relevant guidelines and regulations.

### Participants

We reviewed the medical records of patients with exudative AMD from three institutions (University of Chicago, Northwestern University, and Hyogo Medical University) between January 2016 and December 2020. We compared the demographics and clinical characteristics of the two cities: Chicago and Nishinomiya. We also compared results across racial and ethnic categories. The racial and ethnic categories were based on the definition provided by the NIH *All of Us* Research Program. Data. This study included adults in the USA who self-reported as Black or White and adults in Japan who self-reported as Japanese. Patients who had previously undergone intravitreal treatment, including anti-VEGF agents or any type of posterior-segment laser, including PDT, before baseline visits were excluded from this study. Eyes with retinal conditions other than PCV, presence of significant media opacity, or any history of ocular trauma or surgery within three months before baseline visit were also excluded. Within the treatment-naïve exudative AMD group, PCV was diagnosed based on the presence of a polypoidal lesion(s), as observed on fundus examination andfeatures of OCT images. The diagnosis of AMD was made using The Age-Related Eye Disease Study (AREDS) classification^42^. The diagnosis of PCV was based on the modified criteria according to PCV workup group. Color fundus photos and OCT images must have 3 diagnostic criteria: orange nodule color fundus photos, sub-retinal pigment epithelium ring-like lesion, and sharp-peaked pigment epithelial detachment^24,25^. Two independent masked observers (HF and GB) screened the baseline images to determine eligibility. Cases of disagreement were adjudicated by a senior observer (AAF). If the fellow eyes had been diagnosed with treatment naïve PCV without exudative changes, only the eye presenting with exudative changes was included.

### Data collection and image analysis

Data were extracted from medical records at the three hospitals and compiled at the data center in the Department of Ophthalmology, Northwestern University. We analyzed patient demographic characteristics, including age, sex, BMI, smoking status, and systemic *comorbidities* (diabetes mellitus, hypertension, ischemic cardiovascular disease, and end-stage renal disease). We also analyzed clinical data and images. At baseline presentation, VA, OCT, and color fundus photography examination were collected. The VA was measured using a Landolt C chart or Snellen Chart. The OCT equipment used was Spectralis HRA (Heidelberg-Engineering, Heidelberg, Germany) or Cirrus OCT (Carl Zeiss Meditec, Dublin, CA, USA). The location of polypoidal lesion was recorded as either peripapillary (located with one disc diameter from the margins of the disc), macular (located within the area 5.5 mm diameter from the center of the fovea, except for the peripapillary zone), or other location if neither of these locations was applied. If patients were followed for at least one-year, subsequent visits were reviewed. VA and OCT data were collected in these patients at 3-, 6-, and 12-month follow-ups. Anti-VEGF treatment was administered intravitreally either as ranibizumab 0.5 mg/0.05 ml, aflibercept 2 mg/0.05 ml, or bevacizumab 1.25 mg/0.05 ml. PDT with verteporfin was administered with a 6 mg/m^2^ infusion of verteporfin over 10 min, followed by laser delivery at 689 nm 15 min after the start of the infusion. Anti-VEGF injection frequency (pro-re-nata, treat and extend, and fixed dosing) and combination with PDT were based on each hospital’s physician and patient preferences.

Image analysis was performed by the data center at the Department of Ophthalmology, Northwestern University. Two reviewers (HF and GB) analyzed all the images of patients diagnosed with treatment-naïve exudative PCV, and discrepancies were resolved by consensus. If consensus could not be reached, a third observer interpreted the result (AAF). We reviewed baseline color fundus photography and OCT images.

Color fundus photos were reviewed for the following findings: (1) presence of hard exudates identified as small white or yellowish-white deposits with sharp margins, located typically surrounding zones of polypoidal lesions. (2) presence of soft drusen defined as round, confluent, yellow-white deposits with ill-defined borders. (3) presence of subretinal hemorrhage. (4) presence of pachydrusen defined as large sized drusen (> 125 um) with irregular outer contour, which could be isolated or scattered^43^.

OCT images were reviewed for the following findings: (1) presence of intraretinal fluid, defined as cysts within the neuroretina on OCT, (2) presence of pachyvessels as determined by the appearance of large choroidal vessels (pathologically dilated Haller’s vessels) accompanied with overlying choriocapillaris attenuation^44^, (3) presence of double-layer sign defined the presence of an irregular low-lying elevation of the RPE from the underlying intact Bruch’s membrane with low internal reflectivity of more than 250 um in the horizontal dimension and less than 100 um height^45^, (4) CFT was measured using the measurement tool built into the OCT machine, (5) SFCT was measured manually as the distance from Bruch’s membrane to the choroidal-scleral junction at the fovea.

### Outcomes

Outcomes included demographic and clinical characteristics in each tertiary referral center. The primary goal was to assess the differences in clinical characteristics among the 3 tertiary centers. The secondary goal was to assess the differences in clinical characteristics among the different racial and ethnic groups (Black, White, and Japanese). We also evaluated the treatment responses.

### Statistical analysis

All statistical analyses were performed using JMP Pro (version 15.2.0, SAS Institute Inc., Cary, NC, USA). VA was measured with a Landolt C acuity chart or Snellen chart and then converted to a logarithm of the minimum angle of resolution (logMAR) units for statistical analyses. We used Fisher’s exact test, Pearson’s chi-square test, and Mann–Whitney *U* test to compare the demographic and clinical characteristics of the Chicago and Nishinomiya groups. We used Fisher’s exact and Kruskal–Wallis tests to compare the demographic and clinical characteristics differences among three racial and ethnic groups. The Wilcoxon signed-rank test was used to compare the VA, CFT, and SFCT at baseline and after treatment. Univariate linear regression analyses were performed to examine the associations between demographic factors and subretinal hemorrhage. Factors that had a p-value less than 0.1 in the univariate were included in the multivariable linear regression model. p < 0.05 was considered statistically significant.

## Supplementary Information

Supplementary Legends.

Supplementary Figure 1.

Supplementary Table 1.

Supplementary Table 2.

Supplementary Table 3.

Supplementary Table 4.

## Data Availability

The datasets used and/or analyzed during the current study are available from the corresponding author on reasonable request.
