# Supplementary material for: Clinical variations of polypoidal choroidal vasculopathy: A cohort study from Japan and the USA
Source: Sci Rep. 2023 Mar 23;13:4800. doi: 10.1038/s41598-023-31649-x (PMC10036559; doi:10.1038/s41598-023-31649-x)
Supplement: Supplementary file 1 — Supplementary Legends. [file 41598_2023_31649_MOESM1_ESM.docx]

Supplementary Fig 1. Time course of visual acuity (VA), central foveal thickness (CFT), and subfoveal choroidal thickness (SFCT) s by racial and ethnic subgroups.

Vertical bars represent mean ± standard error.
