# Supplementary figures and images for: Clinical variations of polypoidal choroidal vasculopathy: A cohort study from Japan and the USA
Source: Sci Rep. 2023 Mar 23;13:4800. doi: 10.1038/s41598-023-31649-x (PMC10036559; doi:10.1038/s41598-023-31649-x)

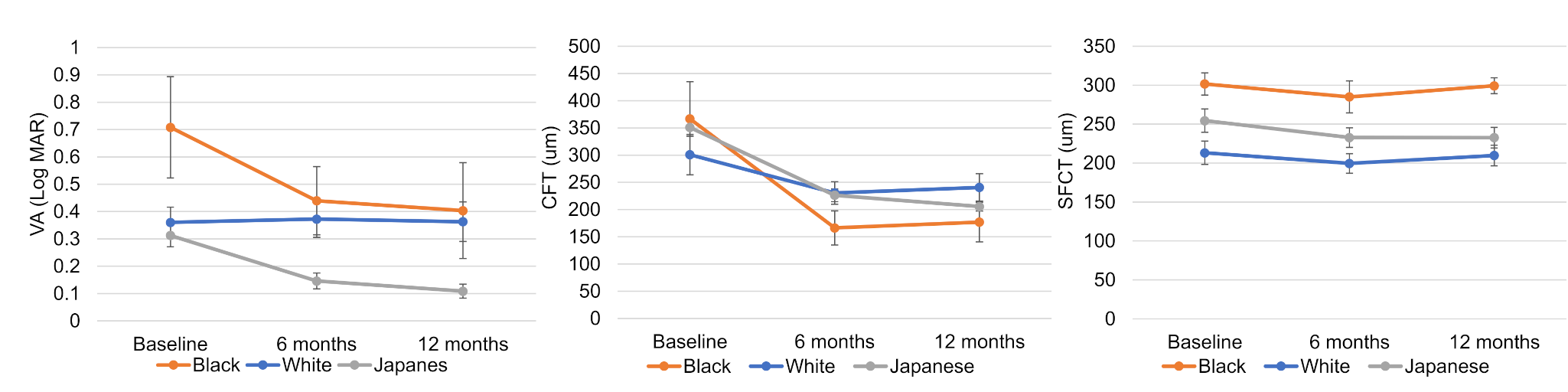

Supplement: Supplementary file 2 — Supplementary Figure 1. [file 41598_2023_31649_MOESM2_ESM.tiff]
