# Supplementary material for: Clinical variations of polypoidal choroidal vasculopathy: A cohort study from Japan and the USA
Source: Sci Rep. 2023 Mar 23;13:4800. doi: 10.1038/s41598-023-31649-x (PMC10036559; doi:10.1038/s41598-023-31649-x)
Supplement: Supplementary file 3 — Supplementary Table 1. [file 41598_2023_31649_MOESM3_ESM.docx]

Supplementary Table 1. The baseline ocular clinical characteristics for Chicago and Nishinomiya patients followed for more than 1 year.

|  | Total  (n=97) | Chicago  (n = 38) | Nishinomiya  (n = 59) | P |
| --- | --- | --- | --- | --- |
| Eyes (Right) (%) | 39 (40.2%) | 19 (50.0%) | 20 (33.9%) | 0.12^a^ |
| Location of polypoidal lesion |  |  |  | 0.19 ^b^ |
| Macular (%) | 71 (73.2%) | 27 (71.1%) | 44 (74.6%) |  |
| Peripapillary (%) | 23 (18.6%) | 10 (26.3%) | 8 (13.6%) |  |
| Other (%) | 8 (8.2%) | 1 (2.7%) | 7 (11.9%) |  |
| VA | 0.407 ± 0.410 | 0.554 ± 0.491 | 0.312 ± 0.319 | 0.005^c^ |
| Hard exudates (%) | 29 (29.9%) | 9 (28.3%) | 20 (33.8%) | 0.37 ^a^ |
| Soft drusen (%) | 36 (37.2%) | 20 (52.7%) | 16 (27.1%) | 0.017^a^ |
| Pachydrusen (%) | 36 (37.1%) | 11 (28.9%) | 25 (42.3%) | 0.20 |
| Subretinal hemorrhage (%) | 35 (36.1%) | 15 (39.7%) | 20 (33.9%) | 0.66^a^ |
| Intra retinal fluid (%) | 21 (21.6%) | 14 (36.8%) | 7 (11.9%) | 0.005^a^ |
| Pachyvessels (%) | 54 (55.7%) | 16 (42.1%) | 38 (64.4%) | 0.038^a^ |
| Double-layer sign | 54 (55.7%) | 17 (44.7%) | 37 (62.7%) | 0.097 |
| CFT (um) | 338 ± 145 | 319 ± 174 | 351 ± 122 | 0.07^c^ |
| SFCT (um) | 226 ± 79 | 235 ± 75 | 254 ± 110 | 0.75^c^ |

^a^ Fisher’s exact test, ^b^ Pearson’s chi-square test and ^c^ Mann–Whitney U test were used to calculate p values.

*Significant at P < 0.05.

Abbreviations: VA, visual acuity; CFT, central foveal thickness; SFCT, subfoveal choroidal thickness; AMD, age-related macular degeneration.
