# Supplementary material for: Clinical variations of polypoidal choroidal vasculopathy: A cohort study from Japan and the USA
Source: Sci Rep. 2023 Mar 23;13:4800. doi: 10.1038/s41598-023-31649-x (PMC10036559; doi:10.1038/s41598-023-31649-x)
Supplement: Supplementary file 4 — Supplementary Table 2. [file 41598_2023_31649_MOESM4_ESM.docx]

Supplementary Table 2. Baseline demographic and systemic characteristics comparing Black, White, and Japanese individuals.

|  | Total  (n = 119) | Black  (n = 18) | White  (n = 21) | Japanese  (n = 80) | P |
| --- | --- | --- | --- | --- | --- |
| Age (years) | 74.1 ± 9.3 | 76.8 ± 7.8 | 70.3 ± 14.0 | 74.3 ± 8.2 | 0.21^a^ |
| Gender (Male) (%) | 73 (59.3%) | 2 (9.5%) | 6 (33.3%) | 65 (77.4%) | < 0.001^b^ |
| BMI | 24.6 ± 4.5 | 27.1 ± 5.7 | 29.5 ± 4.3 | 23.1 ± 3.0 | < 0.001^a^ |
| Smoker (Current or former) (%) | 61 (49.6%) | 12 (57.1%) | 11 (61.1%) | 38 (45.2%) | 0.42 ^b^ |
| DM (%) | 17 (13.8%) | 2 (9.5%) | 5 (27.8%) | 10 (11.9%) | 0.18^b^ |
| HTN (%) | 75 (60.9%) | 11 (52.3%) | 16 (88.8%) | 48 (57.1%) | 0.02^b^ |
| Ischemic cardiac disease (%) | 11 (8.9%) | 0 (0%) | 2 (11.1%) | 9 (10.7%) | 0.31^b^ |
| Renal disease (%) | 3 (2.4%) | 0 (0%) | 2 (11.1%) | 1 (1.2%) | 0.08^b^ |

^a^ Kruskal-Wallis test and ^b^ Fisher’s exact test were used to calculate p values.

*Significant at P < 0.05.

Abbreviations: BMI, body mass index; DM, diabetes mellitus; HTN, hypertension.
