# Supplementary material for: Clinical variations of polypoidal choroidal vasculopathy: A cohort study from Japan and the USA
Source: Sci Rep. 2023 Mar 23;13:4800. doi: 10.1038/s41598-023-31649-x (PMC10036559; doi:10.1038/s41598-023-31649-x)
Supplement: Supplementary file 5 — Supplementary Table 3. [file 41598_2023_31649_MOESM5_ESM.docx]

Supplementary Table 3. The baseline clinical ocular characteristics among Black, White, and Japanese.

|  | Total (n=119) | Black (n = 18) | White (n = 21) | Japanese (n = 80) | P |
| --- | --- | --- | --- | --- | --- |
| Eyes (Right) (%) | 47 (39.5%) | 8 (44.4%) | 11 (52.4%) | 28 (35.0%) | 0.32^a^ |
| Location of polypoidal lesion |  |  |  |  | 0.08^a^ |
| Macular (%) | 86 (72.3%) | 10 (55.6%) | 18 (85.7%) | 58 (72.5%) |  |
| Peripapillary (%) | 22 (18.5%) | 7 (38.9%) | 3 (14.3%) | 12 (15.0%) |  |
| Other (%) | 11 (9.2%) | 1 (5.6%) | 0 (0%) | 10 (12.5%) |  |
| VA | 0.395 ± 0.434 | 0.793 ± 0.705 | 0.366 ± 0.244 | 0.312 ± 0.340 | 0.008^b^ |
| Hard exudates (%) | 40 (33.6%) | 10 (55.6%) | 3 (14.3%) | 27 (33.8%) | 0.03^a^ |
| Soft drusen (%) | 41 (34.5%) | 6 (33.3%) | 15 (71.4%) | 20 (25.0%) | 0.005^a^ |
| Pachydrusen (%) |  |  |  |  |  |
| Subretinal hemorrhage (%) | 52 (43.7%) | 13 (72.0%) | 7 (33.3%) | 32 (40.0%) | 0.03^a^ |
| Intra retinal fluid (%) | 27 (22.7%) | 5 (27.8%) | 10 (47.6%) | 12 (15.0%) | 0.006^a^ |
| Pachyvessels (%) | 67 (54.5%) | 11 (61.1%) | 6 (28.6%) | 50 (62.5%) | 0.02^a^ |
| Double-layer sign |  |  |  |  |  |
| CFT (um) | 341 (145) | 372 (207) | 302 (161) | 336 (27) | 0.32^b^ |
| SFCT (um) | 247 (100) | 275 (62) | 211 (71) | 247 (115) | 0.11^b^ |
| Fellow eye |  |  |  |  |  |
| Pachychoroid disease (%) | 26 (21.8%) | 4 (22.2%) | 4 (19.0%) | 18 (22.5%) |  |
| AMD (%) | 15 (12.6%) | 1 (5.6%) | 6 (28.6%) | 8 (10.0%) |  |
| CFT (Fellow eye) (um) | 200 (52) | 198 (57) | 203 (50) | 200 (57) | 0.93 |
| SFCT (Fellow eye) (um) | 223 (86) | 213 (65) | 183 (70) | 237 (91) | 0.05 ^b^ |

^a^ Kruskal-Wallis test and ^b^ Fisher’s exact test were used to calculate p values. *Significant at P < 0.05.

Abbreviations: VA, visual acuity; CFT, central foveal thickness; SFCT, subfoveal choroidal thickness; AMD, age-related macular degeneration.
