# Supplementary material for: Clinical variations of polypoidal choroidal vasculopathy: A cohort study from Japan and the USA
Source: Sci Rep. 2023 Mar 23;13:4800. doi: 10.1038/s41598-023-31649-x (PMC10036559; doi:10.1038/s41598-023-31649-x)
Supplement: Supplementary file 6 — Supplementary Table 4. [file 41598_2023_31649_MOESM6_ESM.docx]

Supplementary Table 4. Treatment approach and number of anti-vascular endothelial growth factor (anti-VEGF) injections visits 1 year by racial and ethnic background.

|  | Total  (n = 90) | Black  (n = 11) | White  (n=20) | Japanese  (n = 59) | P |
| --- | --- | --- | --- | --- | --- |
| Treatment |  |  |  |  | < 0.001^b^ |
| Anti-VEGF monotherapy | 56 (59.6%) | 19 (95.0%) | 10 (90.9%) | 27 (42.9%) |  |
| Combination therapy | 38 (40.4%) | 1 (5.0%) | 1 (9.1%) | 36 (57.1%) |  |
| Number of Anti-VEGF (1 year) |  | 8.2 ± 3.6 | 6.8 ± 3.3 | 3.8 ± 2.4 | < 0.001^a^ |

^a^ Kruskal-Wallis test and ^b^ Fisher’s exact test were used to calculate p values. *Significant at P < 0.05.
